# Knowledge of Stroke Risk Factors among Stroke Survivors in Nigeria

**DOI:** 10.1155/2016/1902151

**Published:** 2016-11-01

**Authors:** Grace Vincent-Onabajo, Taritei Moses

**Affiliations:** Department of Medical Rehabilitation (Physiotherapy), College of Medical Sciences, University of Maiduguri, PMB 1069, Maiduguri, Borno State, Nigeria

## Abstract

*Background*. Knowledge of stroke risk factors is expected to reduce the incidence of stroke—whether first-ever or recurrent. This study examined knowledge of stroke risk factors and its determinants among stroke survivors.* Methods*. A cross-sectional survey of consenting stroke survivors at two physiotherapy facilities in Nigeria was carried out. Sociodemographic and clinical data were obtained and knowledge of stroke risk factors (defined as the ability to mention at least one correct risk factor) was assessed using open-ended questionnaire. Data were treated with descriptive statistics and logistic regression analysis.* Results*. Sixty-nine stroke survivors (male = 72.5%; mean ± SD age = 49.7 ± 10.6 years) participated in the study. Thirty-four (49.4%) participants had knowledge of stroke risk factors. Only educational level was significantly associated with knowledge and participants with tertiary educational qualification were about 48 times (odds ratio = 48.5; CI = 7.6–309.8; *P* < 0.0001) more likely to be knowledgeable than those with no education.* Conclusion*. Less than half of the participants had knowledge of stroke risk factors. Participants with tertiary education were significantly more knowledgeable than those with lower educational qualifications. Effective means of educating stroke survivors on stroke risk factors should be identified and adopted.

## 1. Introduction

Stroke is a condition that is known to affect virtually every aspect of the survivor's life. Adverse consequences on the physical, psychological, emotional, social, and economic status of stroke survivors place stroke among the leading causes of diminished quality of life globally [1–3]. Preventing stroke thus becomes a critical, better, and keenly sought after option. While prevention of stroke is crucial for all, individuals who have suffered a previous stroke are at increased risk of recurrence [4, 5]; hence secondary prevention becomes a major concern. Recurrent stroke, although known to be characterized by greater level of mortality and morbidity compared to first-ever stroke, is preventable [6, 7]. Consequently, guidelines for stroke management identify secondary prevention of stroke as one of the major goals of stroke care [8] while specific guidelines also exist for prevention of recurrent stroke [9].

Secondary prevention of stroke is possible through several means with knowledge of stroke risk factors prominent among effective means of achieving prevention. Several studies have assessed knowledge of stroke risk factors among stroke survivors [10–14]. It could be assumed that, with the suddenness of a stroke event and the accompanying consternation and shock, many stroke survivors and their relatives would try to make sense of their experience by seeking information about the disease and, by so doing, acquire vital knowledge on stroke including its risk factors. Furthermore, stroke survivors who access orthodox health care are expected to receive some information on stroke prevention from their healthcare providers which should enhance their knowledge about stroke. Findings from studies that examined knowledge of stroke risk factors among stroke survivors however showed that majority of stroke survivors had poor knowledge of stroke risk [10–12, 14].

Existing studies on knowledge of stroke risk factors in Nigeria have focused on individuals diagnosed with hypertension and diabetes [15], on general populations [16], and on the population in an educational institution [17], while there is a dearth of information on stroke survivors' knowledge of stroke risk factors. With the prevalence of recurrent stroke in Nigeria [18], information on how knowledgeable stroke survivors are about stroke risk factors is of utmost importance. This is particularly so as such information would assist in devising feasible strategies to enhance knowledge and consequently aid in effective prevention of recurrent stroke. This study therefore surveyed stroke survivors at two facilities in Nigeria with the aim of assessing their knowledge of stroke risk factors. It is expected that data from the study would provide insight into how equipped stroke survivors are with information capable of protecting them from a recurrent stroke. A second aim was to explore personal factor(s) that independently determined knowledge among the stroke survivors.

## 2. Methods

Sixty-nine consenting stroke survivors participated in the study out of 83 stroke survivors available at the study sites during the study period giving a participation rate of 83.1%. The remaining 12 stroke survivors expressed their unwillingness to participate in the study and were therefore excluded while 2 others provided incomplete information which was unusable. In addition to expression of willingness to participate in the study through provision of informed consent, the other inclusion criterion was being an adult (≥18 years of age). All the participants were able to communicate verbally in either English and/or Hausa (a* lingua franca* in Northern Nigeria) languages and were recipients of poststroke physiotherapy at two government hospitals in Maiduguri, the capital of Borno State, Nigeria.

### 2.1. Study Instrument

A modified version of the questionnaire used in a study by Vincent-Onabajo et al. [15] was used to obtain all relevant data in this study. The first part of the questionnaire contained the introductory letter and informed consent form. The second part contained both open and close-ended questions which were used to obtain the sociodemographic and clinical data of the participants. The data were age, gender, educational level (tertiary/college education; secondary/high school education; primary/elementary education), comorbidity (hypertension or diabetes or both), family history of stroke, and poststroke duration.

The third part of the questionnaire contained an open-ended question which required the participants to mention the stroke risk factors they knew. Ability to mention at least one correct stroke factor was considered as “knowledge” while mention of an incorrect factor or inability to mention any factor was regarded as “no knowledge.” A list of stroke risk factors was compiled from existing stroke literature [9, 19]. The list included both well documented and less well documented risk factors and they are age (increasing age), gender (use of oral contraceptives, pregnancy, history of preeclampsia/eclampsia or gestational diabetes, smoking, and postmenopausal hormone therapy may increase stroke risk in women), low birth weight, race/ethnicity (Black, Hispanics), family history of stroke (genetic disposition), hypertension, diabetes, exposure to cigarette smoke, atrial fibrillation and other cardiac conditions, dyslipidemia, carotid artery, stenosis, sickle cell disease, postmenopausal hormone therapy, poor diet (including high salt intake), physical inactivity, obesity and body fat, metabolic syndrome, excessive alcohol consumption, drug abuse, use of oral contraceptives, sleep-disordered breathing, migraine, hyperhomocysteinemia, elevated lipoprotein, hypercoagulability, inflammation, and infection.

### 2.2. Procedure

Ethical approval for the study was obtained from the relevant institutional Research Ethics Committee. Participants were approached at the physiotherapy facilities where the study was conducted after their physiotherapy sessions. All data were collected by the second author and a research assistant through face-to-face interview from February to April 2016.

### 2.3. Statistical Analyses

All sociodemographic, clinical, and knowledge of stroke data were presented with descriptive statistics of mean, standard deviation, frequencies, and percentages as appropriate.

Logistic regression analysis (“enter” method) was used to identify sociodemographic and clinical factors that were significantly associated with knowledge of stroke risk factors. Knowledge of stroke risk factors (knowledge [yes] = 1; no knowledge [no] = 0) was the dependent variable while the independent variables were age, gender, educational level, family history of stroke, and poststroke duration. Level of statistical significance was set at alpha equals 0.05.

## 3. Results

Participants were 50 male (72.5%) and 19 (27.5%) female stroke survivors whose mean ± SD age was 49.7 ± 10.6 years. Details of the sociodemographic and clinical characteristics of the participants are presented in Table 1.

### 3.1. Knowledge of Stroke Risk Factors

Thirty-four (49.3%) participants were able to mention at least one correct stroke risk factor. Hypertension was the most (39.1%) known risk factor (Table 2). An appreciable proportion of the participants also mentioned various incorrect factors such as “thinking” (46.4%) and “stress” (23.2%) (Table 3). Seventeen (24.6%) participants could however not mention any risk factor whether correct or incorrect.

### 3.2. Factors Associated with Knowledge

Educational level was the only significant (*P* < 0.05) and independent determinant of knowledge of stroke risk factors. Compared to other educational levels, participants with tertiary education were found to be 48 times (odds ratio = 48.5; CI = 7.6–309.8; *P* < 0.0001) more likely to be knowledgeable about stroke risk factors compared to those without any education (Table 4).

## 4. Discussion

Knowledge of stroke risk factors is expected to substantially contribute to secondary stroke prevention. Less than half (49.4%) of the participants in this study however knew at least one correct stroke factor. This finding is particularly worrisome especially as all the participants were actively undergoing stroke care at the time of the study. A previous study also reported a 43% knowledge rate among Norwegians diagnosed with stroke/Transient Ischaemic Attack (TIA) [12].

As with other studies of stroke survivors [12–14], hypertension was the most commonly (39.1%) identified stroke risk factor in this study with obesity and consumption of fatty foods coming at a very distant 2nd at 5.8%. Modifiable risk factors such as smoking [13, 14] and alcohol consumption [14] which was among the best known stroke risk factors in previous studies were little known in this present study. In fact, alcohol intake and smoking along with nonmodifiable risk factors such as family history of stroke and older age were the least mentioned by the participants.

There were several misconceptions about stroke risk factors among the study participants. Prominent among them was “thinking,” which was mentioned as a risk factor for stroke by 46.4% of the participants, thereby constituting the most mentioned factor, even higher than hypertension. The expression “thinking” in Nigerian parlance depicts negative/depressive thoughts and worry, and it is widely regarded as a major cause of high blood pressure and cardiac disorders. It is therefore not completely surprising that many of the participants linked “thinking” with stroke. The same explanation may equally apply to the 23.2% that mentioned “stress” as a stroke risk factor. It is also important to note that about one in every four (24.6%) participants could not mention any stroke risk factor, whether correct or otherwise. An important deduction that can be made from these findings is that a significant dearth of appropriate information on stroke risk factors exists among stroke survivors and this may have dire implications for successful secondary prevention of stroke.

Among the sociodemographic characteristics of the participants, only level of education emerged as a significant and independent determinant of knowledge of stroke risk factors. Participants with tertiary educational qualification were knowledgeable of stroke risk factors compared to those with primary (elementary), secondary, or no formal education. It is however important to note that fewer people in Nigeria possess tertiary educational qualifications. Effective means of providing nationwide intensive education on stroke risk factors especially to individuals with lower levels of education should therefore be urgently identified and adopted in the country. For instance, information on stroke prevention should be simple and easy to understand and should be available in indigenous languages using the mass media. The fact that the study is hospital-based would however require cautious interpretation of findings and future community-based studies would be required to provide a more realistic picture and consequently present more generalizable findings. Association between stroke survivors' educational level and their knowledge of stroke risk factors has however been reported in previous studies [11, 14]. Age has also been reported to be associated with knowledge of stroke risk factors among stroke survivors [11, 12] but this had no significant impact on knowledge in this study.

None of the clinical characteristics assessed in the study had any significant association with knowledge of stroke risk factors. Being hypertensive or diabetic (or both) did not influence knowledge and neither did poststroke duration nor family history of stroke. These findings may signify that the specific opportunities created by the above mentioned clinically relevant data were probably not maximized. For instance, having a diagnosis of diabetes and/or hypertension should have provided opportunities for those with these diagnoses to receive information on the role of their diagnoses as stroke risk factors. It is especially important to mention that 84.1% of the participants were known hypertensives while 7.2% were both diabetic and hypertensive yet only 39.1% and 2.9% of the participants, respectively, identified hypertension and diabetes as risk factors for stroke. This scenario is however different from the findings of a previous study in Nigeria which found that 64.3% of persons diagnosed with hypertension and 56.8% of those with diabetes were aware that their conditions were risk factors for stroke while 80% of those with hypertension, diabetes, or a combination of both diseases identified hypertension as a stroke risk factor [15]. The fact that being diagnosed with these common stroke risk factors did not confer any special privilege to affected individuals in terms of knowledge of stroke risk factors among participants in this present study is worrisome. Health professionals involved in the management of persons at risk of stroke, be it first-ever or recurrent stroke, would therefore need to actively include education on stroke prevention in the overall care they provide.

As mentioned above, time from stroke onset (poststroke duration) and having a family history of stroke appeared not to have any influence on whether persons with stroke were knowledgeable about its risk factors or not. A previous study on knowledge of stroke warning signs among stroke survivors showed that clinically related factors, which would have been expected to influence knowledge, such as admission in stroke units, length of hospital stay, postdischarge rehabilitation, and poststroke hospital/clinic visits, had no such impact [20].

While it is widely acknowledged that prevention of stroke is better and cheaper than cure especially in resource-limited settings like Nigeria, the findings of this study showed that an important ingredient in stroke prevention, that is, knowledge of stroke risk factors, is deficient. Health education which is considered pivotal in stroke prevention is reported to have suboptimal uptake in stroke care even in developed and high-income countries [21]. In Nigeria, a recent study showed that health professionals involved in stroke care experienced constraints in providing adequate information on secondary stroke prevention to stroke survivors and their caregivers especially because of their heavy workload and the additional time that might be involved [22]. The authors of that study however suggested that having independent stroke support groups to address such issues might help the situation. In all, workable, feasible, culturally sensitive methods of providing and disseminating information about stroke especially its risk factors are urgently required. Future trials on the effectiveness of such methods are therefore expedient.

### 4.1. Limitations of the Study

The small sample size in this study is a major limitation which affected the outcome of the logistic regression analysis and resulted in the wide confidence intervals obtained. It is however important to mention that all the stroke survivors available during the study period were approached and 83.1% of them participated in the study. The use of open-ended questions to obtain the risk factors known by participants could also have affected the outcome of the study since previous studies have reported a poorer performance in identifying stroke risk factors with open-ended questions compared to close-ended ones [23]. However, open-ended questions are seen to better reflect the knowledge base of respondents and eliminate guess work.

## 5. Conclusion

Recurrent stroke and its debilitating consequences can and should be prevented. The findings of this study however suggested that a lot is lacking in terms of knowledge of stroke survivors about the risk factors of stroke, a situation which will no doubt adversely affect the potential to prevent the disease. The misconceptions about what constitutes stroke risk factors are also particularly worrisome. Healthcare professionals especially those that are directly involved in the care of stroke patients must therefore rise to the occasion. Similarly, the government and relevant policymakers must see the situation as a call to action by putting in place necessary policies that will facilitate and enhance stroke education. Furthermore, individuals with little or no education should be given special consideration when developing strategies and programmes geared towards providing information on, and improving awareness of, stroke risk factors.

## Figures and Tables

**Table 1:** Characteristics of the participants (*N* = 69).

Variable	Value	
Age (years)		
Mean ± SD	49.7 ± 10.6	
Range	29–70	
Poststroke duration (months)		
Mean ± SD	16.7 ± 14.5	
Range	0.5–60	
	*f*	%
*Gender*		
Male	50	72.5
Female	19	27.5
*Family history of stroke*		
Positive	14	20.3
Negative	55	79.7
*Educational level*		
None	24	34.8
Primary	3	4.3
Secondary	19	27.5
Tertiary	23	33.3

**Table 2:** Correct risk factors mentioned by participants^*∗*^.

Risk factor	*f*	%
Hypertension	27	39.1
Obesity	4	5.8
Consumption of fatty foods	4	5.8
High salt intake	3	4.3
Hypercholesterolemia	3	4.3
Inactivity	2	2.9
Diabetes	2	2.9
Pregnancy	2	2.9
Family history of stroke	1	1.4
Old age	1	1.4
Excessive alcohol consumption	1	1.4
Cigarette smoking	1	1.4

^*∗*^Participants were allowed to mention as many risk factors as they knew.

**Table 3:** Incorrect risk factors mentioned by participants^*∗*^.

Risk factor	*f*	%
Thinking (negative thoughts)	32	46.4
Stress	16	23.2
Consumption of kola nut	6	8.7
High sugar intake	3	4.3
Anxiety	3	4.3
Anger	1	1.4

^*∗*^Participants were allowed to mention as many risk factors as they knew.

**Table 4:** Logistic regression analysis for determinants of knowledge of stroke risk factors.

Variable	Odds ratio	CI	*P* value
*Age*	0.99	0.93–1.06	0.88
*Gender *			
Male	1.03	0.23–4.44	0.96
Female	1.00	Reference	Reference
*Educational level *			
Primary	1.45	0.06–33.50	0.81
Secondary	3.63	0.87–15.15	0.07
Tertiary	48.49	7.59–309.80	0.000^*∗*^
*Family history of stroke*			
Positive	1.12	0.23–5.45	0.88
Negative	1.00	Reference	Reference
*Poststroke duration*	1.01	0.96–1.07	0.48

CI: confidence interval.

^*∗*^Statistically significant at *P* < 0.001.
